# Emetogenicity of Antibody-Drug Conjugates (ADCs) in Solid Tumors with a Focus on Trastuzumab Deruxtecan: Insights from an Italian Expert Panel

**DOI:** 10.3390/cancers14041022

**Published:** 2022-02-17

**Authors:** Giampaolo Bianchini, Grazia Arpino, Laura Biganzoli, Sara Lonardi, Fabio Puglisi, Daniele Santini, Matteo Lambertini, Giovanni Pappagallo

**Affiliations:** 1 Department of Medical Oncology, IRCCS Ospedale San Raffaele, 20132 Milan, Italy; 2 School of Medicine and Surgery, Vita-Salute San Raffaele University, 20132 Milan, Italy; 3 Department of Clinical Medicine and Surgery, University of Naples “Federico II”, 80131 Naples, Italy; grazia.arpino@unina.it; 4 Medical Oncology Department, Department of Oncology, Hospital Santo Stefano, 59100 Prato, Italy; laura.biganzoli@uslcentro.toscana.it; 5 Oncology Unit 3, Department of Oncology, Veneto Institute of Oncology IRCCS, 35128 Padova, Italy; sara.lonardi@iov.veneto.it; 6 Department of Medicine (DAME), University of Udine, 33100 Udine, Italy; fabio.puglisi@uniud.it; 7 Department of Medical Oncology, Unit of Medical Oncology and Cancer Prevention, Centro di Riferimento Oncologico di Aviano (CRO) IRCCS, 33081 Aviano, Italy; 8 Medical Oncology, University Campus Bio-Medico, 00128 Roma, Italy; d.santini@unicampus.it; 9 Department of Internal Medicine and Medical Specialties (DiMI), School of Medicine, University of Genova, 16132 Genova, Italy; matteo.lambertini@unige.it; 10 Department of Medical Oncology, UOC Clinica di Oncologia Medica, IRCCS Ospedale Policlinico San Martino, 16132 Genova, Italy; 11 School of Clinical Methodology, IRCCS “Sacro Cuore-Don Calabria” Hospital, 37024 Negrar di Valpolicella, Italy; giovanni.pappagallo@gmail.com

**Keywords:** antibody-drug conjugates, antiemetic, nausea, vomiting

## Abstract

**Simple Summary:**

Four antibody-drug conjugates (ADCs) are approved for the treatment of solid tumors, improving the therapeutic index. Despite their high selectivity, nausea and vomiting are the most frequently observed side effects. A deeper understanding of the potential risk for nausea and vomiting is crucial, as they can affect patients’ quality of life and treatment adherence. Prophylaxis with the potential combination of antiemetic therapy with complementary non-pharmacological approaches are even more important, considering that ADC therapies are generally given continuously until disease progression or the occurrence of toxicities.

**Abstract:**

In the past decade, nine antibody-drug conjugates (ADCs) have been approved for the treatment of various tumors, four of which specifically for solid malignancies. ADCs deliver the cytotoxic payload to the cancer site, thereby improving chemotherapy efficacy while reducing systemic drug exposure and toxicity. With their high selectivity, ADCs are associated with a manageable side-effect profile, with nausea and vomiting being among the most frequent toxicities, although this may vary according to the respective ADC and the associated payload. Information about the emetic risk of the new ADC compounds is limited. Three virtual focus groups of Italian oncologists were held to raise awareness on the importance of an antiemetic prophylaxis regimen to prevent and mitigate ADC-associated emesis and its sequelae. After reviewing published evidence and guidelines, the three expert panels shared their experience on the early use of ADCs gained through the participation in specific clinical trials and their clinical practice. The following issues were discussed: antiemetic therapy during trastuzumab deruxtecan treatment, with a protocol adopted at the San Raffaele Hospital (Milan, Italy); the use of steroids; the management of anticipatory nausea during trastuzumab deruxtecan therapy; nutritional counselling; and effective doctor–patient communication. The experts acknowledged that recommendations should be drug-specific, and formulated opinion-based advice intended to guide physicians in their daily practice until further evidence emerges.

## 1. Introduction

Antibody-drug conjugates (ADCs) are potent targeted therapies with proven efficacy across a variety of hematological and solid malignancies [1,2,3,4]. They are composed of a monoclonal antibody that recognizes a specific tumor antigen, an extremely potent cytotoxic drug known as payload, and a linker connecting the payload to the antibody [4].

The considerable progress made in the past decade has led to the approval of 9 ADCs for the treatment of various tumors, and more than 100 are currently in different stages of clinical development [1,2,4,5]. The 4 ADCs approved for the treatment of solid tumors are described in Table 1.

By efficiently delivering the toxic payload to the tumor site, ADCs exploit the targeted agent to improve chemotherapy efficacy while reducing systemic drug exposure and toxicity [10]. With their high selectivity, these compounds are associated with a manageable and tolerable side-effect profile. The mechanisms underlying ADC-induced side effects include low expression of the target antigen on normal cells (off-tumor on-target) and the distribution of the target across specific organs, which may result in ADC-specific toxicities, early cleavage of the linker that causes the release of the payload (which due to its potency is toxic at a very low concentration), and binding of the monoclonal antibody to Fc and mannose receptors, possibly causing off-target toxicities. These mechanisms, as well as the characteristics of the payload, contribute to defining the specific toxicity profile of each ADC [11,12].

Among others, nausea and vomiting are the most frequently observed side effects of anti-cancer therapies. They vary according to the specific ADC administered, but also to patient characteristics such as sex, age, prior experience of emesis during pregnancy, and history of motion sickness [13,14,15]. There are three types of emesis, each one with particular characteristics that require specific antiemetic approaches: (i) acute emesis, which occurs within 24 h of administration; (ii) delayed emesis, which has been arbitrarily defined as emesis presenting more than 24 h after administration, and can persist for several days, even up to the subsequent cycle of treatment; (iii) anticipatory emesis, which occurs immediately before administration of the anti-cancer therapy in patients with prior experience of acute or delayed treatment-induced emesis. It is usually triggered by the sight and/or smell of the room where therapy is administered [13,14,15].

Recommended optimal antiemetic treatment regimens are defined by international guidelines [15,16]. To determine the most appropriate antiemetic regimen, anti-cancer agents are classified into four groups based on their emetogenic potential: high risk (>90%), moderate risk (between 30 and 90%), low risk (between 10 and 30%), and minimal risk (<10%). Although this classification is arbitrary and does not take into account individual risk factors for emesis, it represents a useful clinical reference framework for commonly used drugs and for new compounds.

## 2. Emetic Risk Associated with ADC in Solid Tumors

Reviewing the incidence of this side effect reported for available ADCs, it appeared clear that the incidence of nausea and vomiting is quite variable among new ADCs [6,7,8,9,13,14,15] (Table 2 and Table 3). Some studies separately report only the proportion of patients experiencing a grade 3 event, whereas it would be important to present and distinguish between the proportion of grades 1 and 2, which, instead, are often pooled together.

Grades 1–3 of nausea and vomiting are defined using the Common Terminology Criteria for Adverse Events v5.0 (CTCAE) in Table 4.

From a quantitative and objective point of view, in most studies, the only quantifiable parameter is the number of individuals who do not experience emesis at all. Indeed, side effects are usually reported by the most severe grade recorded for the patient at any time, regardless of symptom duration. To understand and quantify the impact of nausea and vomiting, not only the grade but, maybe more importantly, the duration of the symptom should be taken into account: indeed, persisting nausea, even if of grade 1, might significantly impact a patient’s quality of life and compliance to therapy.

The low and moderate incidence and severity of nausea and vomiting associated with the first ADC approved for solid tumors, trastuzumab emtansine, which does not require antiemetic therapy, has led the oncology community to expect a similar safety profile for other ADCs. The most recent update of the guidelines for nausea and vomiting prevention mentioned the emetic risk for three of the available agents, but did not dedicate particular attention to this class of compounds and did not specifically reinforce the need to adopt antiemetic strategies [13,14,15,19]. In addition, for some of these compounds, antiemetic treatment was not mandated in the early clinical trials and was left to the investigator’s discretion. This might have reduced awareness of the potential for this side effect to affect patients’ quality of life, reducing treatment adherence, leading to premature discontinuation and to detrimental consequences on outcomes [27]. This is highly important considering that, unlike conventional chemotherapies at higher emetic potential, such as anthracyclines which are administered for a limited period, ADC therapies are generally given continuously until disease progression or occurrence of toxicities, with the median treatment duration reaching 18 months in some cases [22]. Therefore, it is highly recommended to use antiemetic therapy from the first ADC therapy administration to ensure patients the maximum benefit, maintaining compliance to treatment while preserving and maximizing the quality of life.

To raise awareness on the importance of appropriate antiemetic therapy to prevent and mitigate ADC-associated emesis and its sequelae, providing oncologists with practical indications to adequately manage it, three virtual focus groups of Italian oncologists were held between November and December 2020. Each focus group included a moderator (GP), two scientific coordinators with experience in the use of ADCs, and experienced breast oncologists. The aim of the focus groups was to formulate expert opinion-based advice for the management of ADC-induced nausea and vomiting [28].

After reviewing published evidence and available guidelines, the three expert panels shared their experience of the early use of ADCs, gained through the participation in specific clinical trials, as well as their clinical practice. 

We report here the outcome and recommendations of the three focus groups.

## 3. Managing ADC-Induced Nausea and Vomiting in Cancer Patients: Results from Three Focus Groups

Due to the wide variation in emetogenic potential of the four ADCs considered, the expert panels agreed that guidance should be drug specific. Experience with trastuzumab-emtansine was the most extensive and there was agreement that limited experience with either enfortumab vedotin or sacituzumab govitecan highlighted the need for further clinical trial evidence for these molecules. General consensus for agreement with current guidelines was reached for all but one of the ADCs (see Table 5). For Trastuzumab deruxtecan it was agreed that there was a need to recommend prophylactic antiemetic therapy, See Section 3.1 below for detailed discussion of the recommendations.

The recent ASCENT trial [26], conducted in metastatic triple-negative breast cancer patients, further supports the use of antiemetic prophylaxis, although it remains to be defined whether the optimal antiemetic regimen requires two or three drugs, considering that the investigators were allowed to choose between the two options and that the data on this side-effect control, according to the regimen used, are missing. The expert panel agreed on the importance of collecting this additional information within the clinical trials and sharing it with the medical community to guide optimization of the side effect management for these new compounds.

According to the direct experience of most of the experts with trastuzumab-deruxtecan (T-DXd) and the ASCO and NCCN recommendations, the experts agreed that T-DXd is the only ADC for which the need for prophylaxis of chemotherapy-induced nausea and vomiting could be discussed in depth.

### 3.1. Antiemetic Therapy for Trastuzumab Deruxtecan (T-DXd)

#### 3.1.1. Protocol for Antiemetic Prophylaxis Adopted at the San Raffaele Hospital (Milano, Italy)

The experts reviewed available data concerning the emetogenic potential of T-DXd (Table 2) and agreed with the ASCO and NCCN guidelines (Table 3) that T-DXd should be classified as associated with moderate emetic risk, and, accordingly, prophylactic therapy should be used [13,16].

However, these guidelines are relatively recent and at the time of the initial clinical development of T-DXd, its emetogenic potential was not well known and the management of this side effect led to investigators’ judgement.

The two major classes of antiemetic drugs are represented by 5-HT3 and NK1 antagonist. The molecular mechanism of these receptors in emesis and the therapeutic role of their antagonist has been described elsewhere [29].

Clinical investigators at the San Raffaele Hospital (Milano, Italy) noticed that 60–65% of patients experienced nausea with or without vomiting during the first cycle and asked for pharmacological management from the second cycle. In addition, patients not experiencing GI side effects during the initial cycles were also in need of receiving an antiemetic prophylaxis at some point. As a result, an antiemetic prophylactic protocol was defined to be applied consistently for all patients. The experts highlight that, based on the present experience, nausea of grades 1–2 tended to persist over time and in subsequent cycles if not optimally controlled.

The protocol defined at the San Raffaele Hospital was presented and discussed during the three expert panels and received a general consensus of agreement from all experts (Table 6).

In the opinion of the three panel groups, a two-drug regimen (DEX + 5-HT_3_) is the most appropriate regimen for the vast majority of patients treated with T-DXd monotherapy. A thorough evaluation of individual patient characteristics and clinical history is crucial to tailor treatment and optimize efficacy while limiting toxicities. In the presence of factors predictive of increased risk of emesis (e.g., characteristics and site of the tumor, patient age and gender, constipation, prior nausea induced by chemotherapy, etc.), the experts agreed that it might be highly appropriate for selected patients to start antiemetic prophylaxis from the first cycle with a three-drug regimen (including NK1 receptor blockers). In the case of anything less than an optimal control of emesis during the first cycle using the DEX + 5-HT_3_ regime, the expert panel discouraged attempts to introduce minor modifications and to fine tune this prophylactic regimen, suggesting instead to immediately escalate treatment for selected patients administered before the second cycle using a three-drug regimen (including NK1 blockers). The experts highlighted that the aim should not simply be to achieve control of emesis; however, an optimal control of this side effect would consider the long duration of the treatment expected with this extremely effective compound, and avoid the risk of discontinuation due to patients’ decisions, improving the quality of life during treatment.

#### 3.1.2. Steroids

Steroids, in particular dexamethasone, play a key role in the management of emesis, especially to prevent delayed emesis. Although generally well-tolerated, dexamethasone exhibits side effects even with short-term use. A study demonstrated that patients receiving 10 or 20 mg dexamethasone before moderately emetogenic chemotherapy and then 4–8 mg from 1 to 3 days post-chemotherapy reported insomnia, gastrointestinal symptoms, agitation, increased appetite, weight gain, and skin rash [30]. To explore whether the frequency of dexamethasone administration could be reduced without losing antiemetic efficacy, a number of studies have been carried out [31,32]. Compared to other drugs included in antiemetic combination regimens, the dose of steroids depends on the type of antiemetic regime used. The experts agreed with the guidelines (NCCN, ASCO) that to reduce some of the most bothersome adverse effects, steroids should be administered as a single dose in the morning. However, the experts believed that 4 mg from day 2 to 3 could be enough for some patients without individual risk factors for nausea, and if no nausea is experienced during the first day of the cycle.

#### 3.1.3. Management of Anticipatory Nausea during T-DXd Therapy

It is well established that there is an important psychological component to the occurrence of nausea [33]. If a patient has already experienced this adverse event, he/she may experience anticipatory and memory-induced nausea or may have chemotherapy-induced bias and be tempted to discontinue therapy with T-DXd. Effective prophylaxis from the first drug administration is the mainstay of treatment for anticipatory nausea, but effective communication and support along with complementary therapies such as acupuncture may help. [33].

#### 3.1.4. Nutrition Counselling

A moderately emetogenic drug administered over a long period may generate problems that are indirectly associated with nausea and vomiting, for example, loss of appetite/anorexia. This condition may occur and rapidly worsen, especially in patients who experience nausea for more than 2 days. It is therefore advisable to include a nutrition counsellor in the multidisciplinary team managing the patient. A recent randomized trial evaluated the effects of nutrition counselling in breast cancer patients receiving adjuvant chemotherapy. The results indicated a significant reduction in the occurrence of chemotherapy-induced nausea and vomiting, and significant improvements in global health status/quality of life, physical functioning, role functioning, emotional functioning, and cognitive functioning [34].

#### 3.1.5. Doctor–Patient Communication

Knowing patients’ perception of the side effects of anticancer therapy is extremely useful in the process of clinical decision-making. Nausea and hair loss are among the most impactful side effects in the patients’ perception [35].

Inadequate doctor–patient communication about nausea is a relevant barrier to optimal management of this debilitating effect. Published data show that effective doctor–patient communication regarding potential adverse events prior to therapy administration reduces the impact of nausea and vomiting on the patient’s quality of life [36]. Communication strategies should serve to encourage patients to share the responsibility for establishing goals of therapy and understanding the risks and benefits of the selected antiemetic regimen, thereby becoming active participants in their cancer care.

The advice provided by experts is summarized in Table 7.

## 4. Expert Opinion Summary

In view of the expanding use of new ADCs in clinical practice, the optimal management of side effects is key to improve patients’ quality of life and avoid undue early discontinuation due to poor tolerability, which could compromise treatment efficacy. In this context, we focused our attention on the emetic risk associated with ADCs. The methodological approach we followed was to organize three virtual expert focus groups to provide practical suggestions for oncologists to help prevent or at least mitigate the occurrence of nausea and vomiting.

Experts agreed that information about the emetic risk for the new ADC compounds is limited. Not only grade, but also duration of symptoms should be carefully taken into account to define the need of antiemetic prophylaxis to improve patient quality of life.

The experts agreed that the current knowledge gap on optimal antiemetic treatment management should be formally explored in prospective studies. However, considering the availability of these compounds in the market, there is an urgent need to define a consistent and pragmatic approach.

When managing patients receiving ADC therapy at moderate/high risk of nausea and vomiting, it is essential to start antiemetic prophylaxis from the first cycle, accounting for patient’s characteristics with a two- or three-drug regimen. If the nausea is not optimally controlled from the beginning, an immediate switch to a combination recommended for highly emetogenic therapies, including NK1inhibitor, is strongly advisable. Indeed, the attempt to slowly escalate antiemetic therapy when optimal control is not achieved during the first cycle should be discouraged to avoid the potential for anticipatory nausea during the following cycles potentially affecting treatment compliance. Antiemetic therapy must be administered continuously, rather than on an as-needed basis. It is essential that both the doctor and the patient understand the importance of prophylaxis and of maintaining it for the entire period as per protocol and guidelines. The impressive therapeutic success of new ADCs is well represented by the long median treatment duration observed in clinical trials [19], which requires raising the bar of emesis control from desirable to optimal to fulfill the promise of these compounds in terms of compliance and efficacy.

## 5. Conclusions

To ensure patients the maximum efficacy from ADC therapy, side effects must be adequately controlled. Nausea and vomiting are frequent in trials but, in the long term, they seriously affect the quality of patients’ life and their adherence to therapy, with detrimental consequences on the outcome. As nausea and vomiting are among the most frequently reported events, antiemetic prophylaxis should be offered from the start of therapy, and should be tailored to each patient’s characteristics. Antiemetic therapy can be combined with complementary non-pharmacological approaches that may play an important role in controlling the psychological component of nausea, mitigating possible episodes of memory-induced anticipatory nausea.

In the future, to raise awareness on this topic among clinicians and nurses, the collection of patient-reported outcomes on the burden of nausea should be publicized.

## Figures and Tables

**Table 1:** The antibody-drug conjugates currently approved by the Food and Drug Administration (with or without EMA approval) for the treatment of patients with solid tumors.

ADC	Target	Payload	Manufacturer	Indication	Approval
Trastuzumab emtansine [6]	HER2	DM1 (microtubule inhibitor)	Genentech Roche	HER2-positive metastatic breast cancer in patients already exposed to trastuzumab.	FDA, 2013 EMA, 2013
Enfortumab vedotin [7]	Nectin-4	MMAE (microtubule inhibitor)	Astellas/ Seagen	Locally advanced or metastatic urothelial cancer in adult patients who have already received a PD-1 or PDL-1 inhibitor and one prior platinum treatment.	FDA, 2019
Trastuzumab deruxtecan [8]	HER2	Deruxtecan (Topoisomerase I inhibitor)	AstraZeneca/ Daiichi Sankyo	Unresectable or metastatic HER2-positive breast cancer in patients who have received 2 or more anti-HER2-targeted regimens. Locally advanced or metastatic HER2-positive gastric or gastroesophageal (GEJ) adenocarcinoma in patients who have received a prior trastuzumab-based regime.	FDA, 2019 EMA, 2020 * FDA, 2021
Sacituzumab govitecan [9]	Trop-2	SN-38 (topoisomerase inhibitor)	Immunomedics/ Gilead Sciences	Triple-negative breast cancer in adult patients who have received at least 2 prior therapies for relapsed or refractory metastatic disease.	FDA, 2020 EMA, 2021

Abbreviations: HER2 human epidermal growth factor receptor 2; MMAE monomethyl auristatin E; PD-1 programmed cell death 1; PD-L1 programmed death ligand-1; FDA Food and Drug Administration; EMA European Medicines Agency; * And several other approvals across other countries.

**Table 2:** Incidence of nausea (all grades and grade ≥ 3) as reported in the summary of product characteristics (SmPc) and pivotal studies.

	Nausea Incidence (%) All Grades	Nausea Incidence (%) Grade ≥ 3	Vomiting Incidence (%) All Grades	Vomiting Incidence (%) Grade ≥ 3
Trastuzumab emtansine				
SmPc [6]	≥25	N/A	≥25	N/A
EMILIA Study [17]	39.2	0.8	19.0	0.8
MARIANNE Study [18]	47.1–52.2	N/A	21.6	N/A
KATHERINE Study [19]	41.6	0.5	N/A	N/A
Enfortumab vedotin				
SmPc [7]	45	3	18	2
EV-101 Study [20]	38	1	N/A	N/A
EV-201 Study [21]	39	2	N/A	N/A
Trastuzumab deruxtecan				
SmPc [8]	79	3	48.7	4.3
DESTINY Breast01 Study [22]	77.7	7.6	45.7	4.3
DESTINY-Gastric01 [23]	63	5	26	0
Sacituzumab govitecan				
SmPc [9]	69	6	45	4
IMMU-132-01 [24]	67	6	49	6
IMMU-132-01 (NSCLC) (10 mg/kg) [25]	78	9	33	2
ASCENT [26]	57	2	29	1

**Table 3:** Emetogenic potential of each ADC according to the ASCO and NCCN guideline [13,16].

	Emetogenic Potential	
	ASCO	NCCN
Trastuzumab emtansine	Low	Low
Enfortumab vedotin	Low	Moderate
Trastuzumab deruxtecan	Moderate	Moderate
Sacituzumab govitecan	na	High emetic risk

na: not available.

**Table 4:** Nausea and Vomiting Grade, CTCAE v5.0.

Nausea
Grade 1: Loss of appetite without alteration in eating habits.
Grade 2: Oral intake decreased without significant weight loss, dehydration or malnutrition.
Grade 3: Inadequate oral caloric or fluid intake: tube feeding, TPN, or hospitalization indicated.
**Vomiting**
Grade 1: Intervention not indicated.
Grade 2: Outpatient IV hydration; medical intervention indicated.
Grade 3: Tube feeding, TPN, or hospitalization indicated.

**Table 5:** Indications on the management of antiemetic prophylaxis from the ASCO guidelines, the SmPc of each ADC, and clinical trials.

ADC	Dosing Schedule	ASCO Guidelines [5]	SmPc	Clinical Trial
Trastuzumab emtansine [6]	The recommended dose is 3.6 mg/kg given as an intravenous infusion every 3 weeks (21-day cycle) until disease progression or unacceptable toxicity, or a total of 14 cycles.	No general prophylaxis recommended. Only for the rare patients experiencing emesis, administration of a 5-hydroxytryptamine receptor (5-HT3) receptor antagonist or a single 8 mg dose of dexamethasone before antineoplastic treatment is recommended (Evidence type: informal consensus, benefits outweigh harms. Evidence quality: low. Strength of recommendation: moderate).	N/A	Not required and not recommended
Enfortumab vedotin [7]	The recommended dose is 1.25 mg/kg (up to a maximum dose of 125 mg) given as an intravenous infusion over 30 min on Days 1, 8, and 15 of a 28-day cycle until disease progression or unacceptable toxicity.	Patients treated with low-emetic-risk antineoplastic therapy should be offered a 5-HT3 receptor antagonist OR dexamethasone (Evidence Type: informal consensus, benefits outweigh harms. Evidence quality: low. Strength of recommendation: moderate).	N/A	Not required and not recommended, but allowed upon investigator’s decision
Trastuzumab deruxtecan [8]	The recommended dosage is 5.4 mg/kg given as an intravenous infusion once every 3 weeks (21-day cycle) until disease progression or unacceptable toxicity.	It is recommended to offer antiemetic prophylaxis as for moderate emetic compound with a two-drug combination of a 5-HT3 receptor antagonist and dexamethasone (day 1) and appropriate treatment for delayed emesis (Evidence type: evidence-based, benefits outweigh harms. Evidence quality: high. Strenght of recommendation: strong).	N/A	Majority of the ongoing studies recommend prophylactic antiemetic agents prior to infusion of T-DXd and on subsequent days. Antiemetics such as 5-HT3 receptor antagonists or Neurokinin-1 (NK1) receptor antagonists and/or steroids (eg, dexamethasone) should be considered and administered in accordance with the prescribing information or institutional guidelines
Sacituzumab govitecan [9]	The recommended dose is 10 mg/kg given as intravenous infusion once weekly on Days 1 and 8 of continuous 21-day treatment cycles until disease progression or unacceptable toxicity.	So far not mentioned in the guidelines	Premedication with a two- or three-drug combination regimen (e.g., dexamethasone with either a 5-HT3 receptor antagonist or an NK-1 receptor antagonist as well as other drugs as indicated) is recommended for the prevention of chemotherapy-induced nausea and vomiting. Withhold Sacituzumab-govitecan doses in case of grade 3 nausea or grade 3–4 vomiting at the time of scheduled treatment administration, and resume with additional supportive measures when resolved to grade ≤ 1.	Strongly recommended with a two- or three-drug combination regimen

**Table 6:** Antiemetic prophylaxis protocol followed for T-DXd administration at the San Raffaele Hospital.

First Cycle: Dexamethasone (DEX) + 5-HT_3_ Receptor Antagonist (DEX + 5-HT_3_ + NK1 Only in Selected Cases)	
Day 1	Dexamethasone 8 mg iv + Palonosetron 0.25 mg iv (or 0.5 mg p.o.) before the infusion (preferred regimen) *
	Dexamethasone 8 mg iv + Ondansetron 8 mg iv (or 8 mg p.o. twice daily) (or other shorter half-life 5-HT_3_ antagonist) before the infusion (alternative regimen) *
Day 2–3	Dexamethasone 4 mg p.o. (or 8 mg p.o.) once daily) +/− Metoclopramide 10 mg tablets (three time daily, required if suboptimal control)) †
**Second cycle and following cycles: DEX + 5-HT3 (if optimal control) or DEX + 5-HT3 + NK1 (if suboptimal control)**	
If optimum control is obtained during the first (or following) cycle, the prophylaxis used is maintained subsequently	
If optimum control was not achieved during the first (or following) cycle, from the second (or following) cycle it is recommended an escalation of the antiemetic regimen as follow (or similar regimens including NK1-receptor antagonist):	
Day 1	Dexamethasone 12 mg iv + 300 mg netupitant and 0.5 mg palonosetron p.o.
Days 2–4	Dexamethasone 8 mg p.o. daily +/− Metoclopramide 10 mg tablets (three time daily if suboptimal control))
**For patients complaining of nausea or vomiting despite escalated prophylaxis with NK1-receptor antagonist**	
Days 1–4	Olanzapine (5–10 mg p.o. once daily)
**For the management of late breakthrough nausea**	
From day 4	Metoclopramide 10 mg tablets (three time daily until resolution) +/− Dexamethasone 4 mg p.o. (daily until resolution)
	Can be treated with benzodiazepines (loraxepam)

* Palonosetron has a longer half-life than other 5-HT3 receptor antagonists (e.g., ondansetron). Although ondansetron and other 5-HT3 receptor antagonists are acceptable options, in the opinion of the experts, palonosetron should be the preferred option for both patients’ convenience and possibly a better control of the symptoms. † Continuing the short half-life 5-HT3 receptor antagonist could be indicated as suggested by international guidelines.

**Table 7:** Experts’ advice on the management of emetic risk linked to ADCs.

TOPIC	ADVICE
Evaluation of emetic risk	Current evidence regarding the management of ADC-associated emesis is scarce and guidelines provide only general information. Emesis is not a class effect, but each molecule harbors a specific risk.
Prophylaxis need *	T-DM1 is classified as an ADC with low emetic risk; no recommendation is provided, and antiemetic therapy should be provided only on an ad hoc basis whenever needed for patients experiencing emesis. Enfortumab vedotin is characterized by limited data on emetic risk and guidelines disagreed on emetic risk classification. The expert panel stated that more information is needed to define optimal treatment management. Sacituzumab govitecan ^§^ should be offered two- or three-drug regimes as prophylactic treatment, as recommended by guidelines and the manufacturer. Fine-tuning to define the best antiemetic regimen is still needed. T-DXd harbors a moderate emetic risk (30–90%). An antiemetic protocol should be given in accordance with guideline recommendations for chemotherapies with moderate emetic risk.
Tailored prophylaxis for ADC with moderate emetic risk	Prophylaxis is always highly recommended, commonly including a two-drug antiemetic regimen. Prophylaxis should be tailored on patient characteristics and clinical history suggesting an increased risk of emesis (e.g., tumor site, gender, prior nausea induced by prior chemotherapy, etc.). In these cases, it might be recommended to start with a three-drug regimen (including NK1 blockers).
Anticipatory nausea	Prophylaxis is highly recommended since the first administration to prevent this event. It might be useful to consider complementary approaches, such as acupuncture, to be added to prophylaxis protocol.
Nutrition counseling	Nutrition counseling might be useful to define nutrition strategies to prevent or correct the loss of appetite/anorexia.

* Protocols are based on the specific risk associated with each ADC as stated by literature, by the manufacturer and clinical practice. ^§^ more information should come at the conclusion of IMMU-132 trial.
